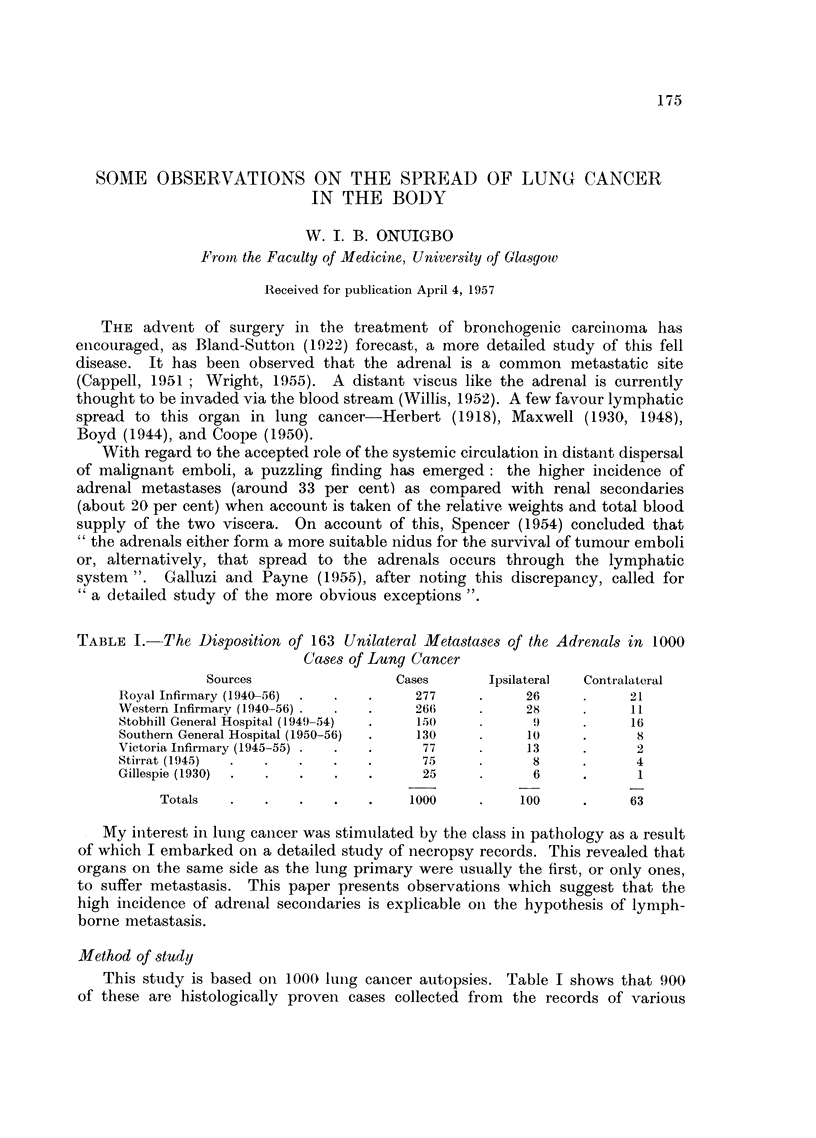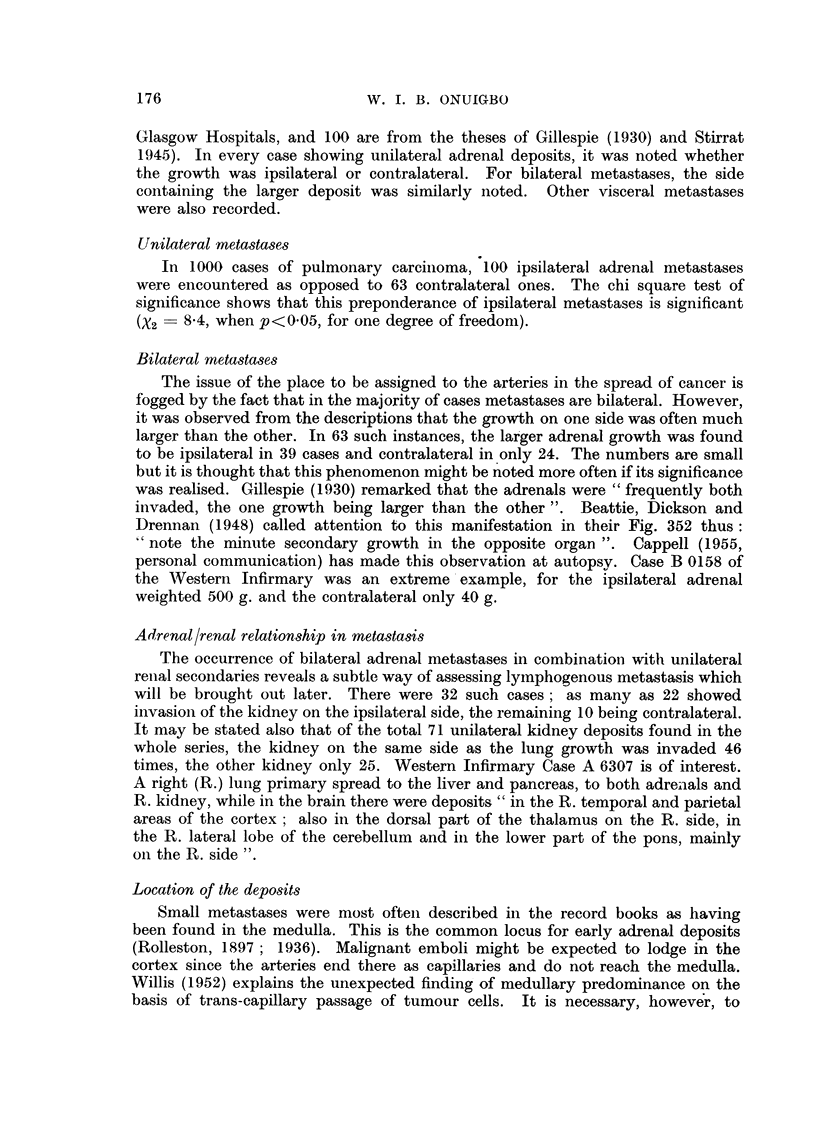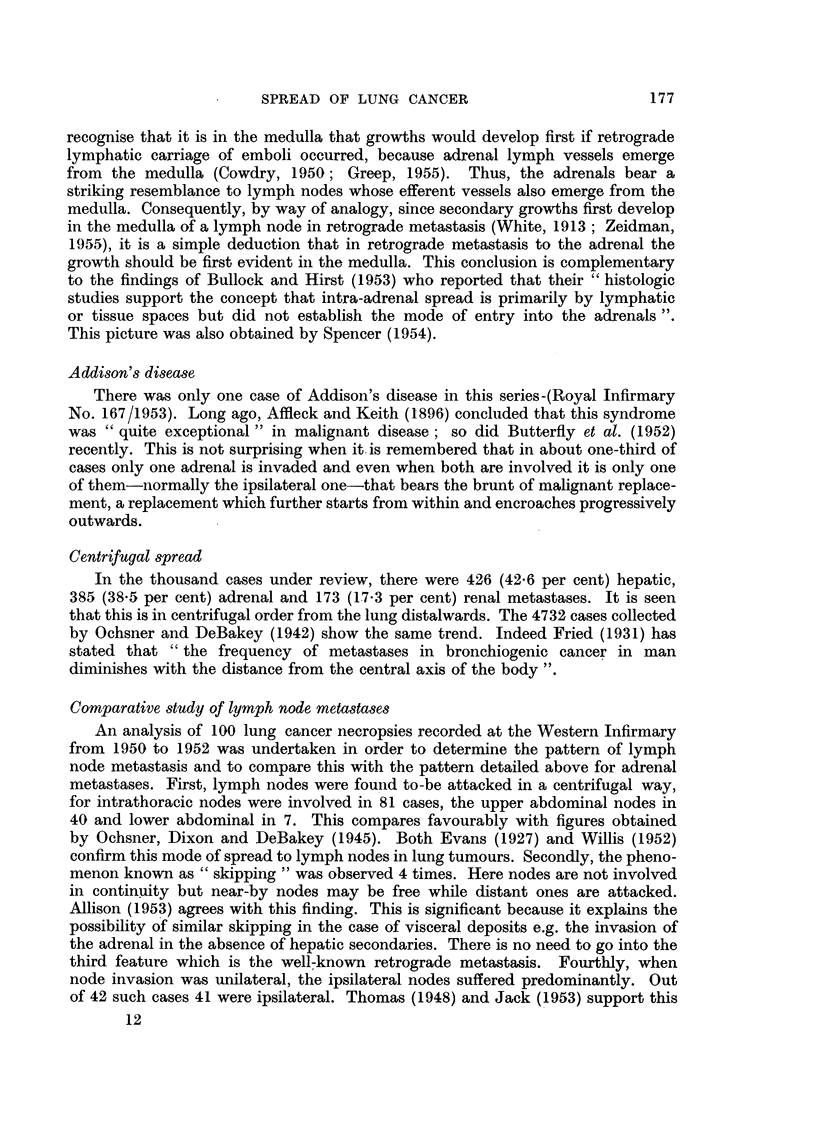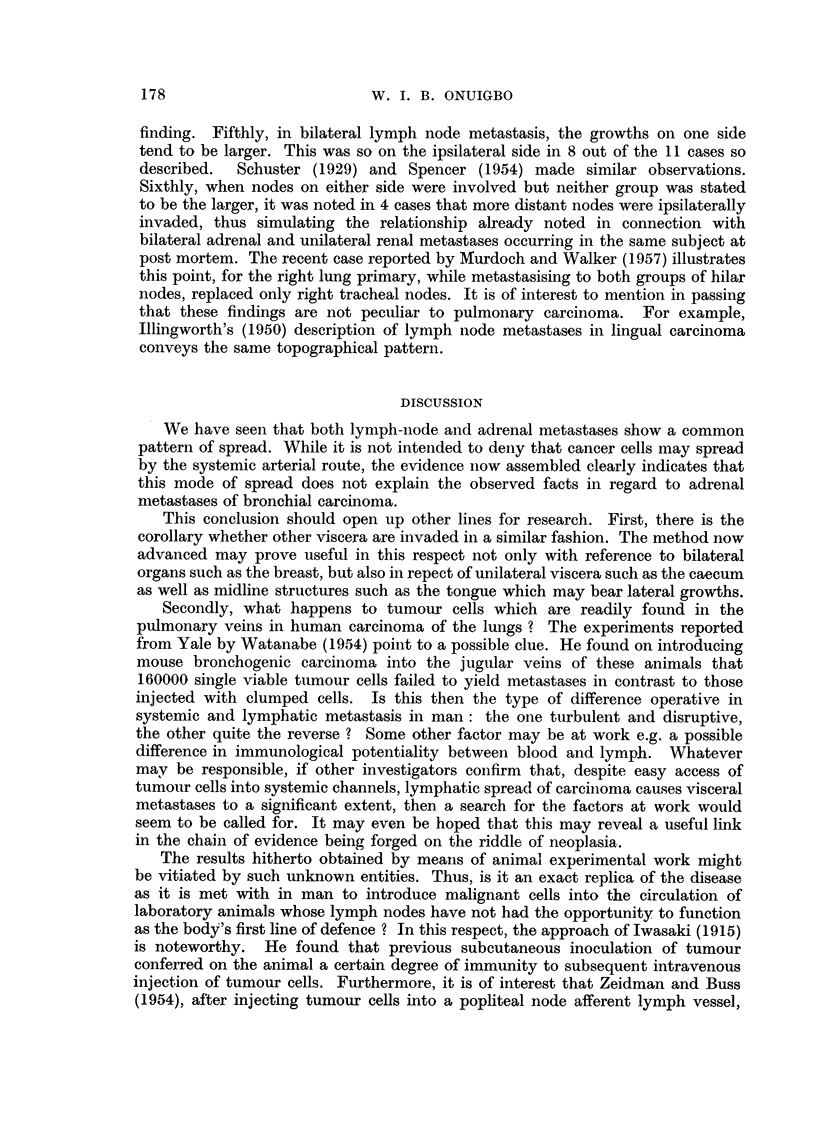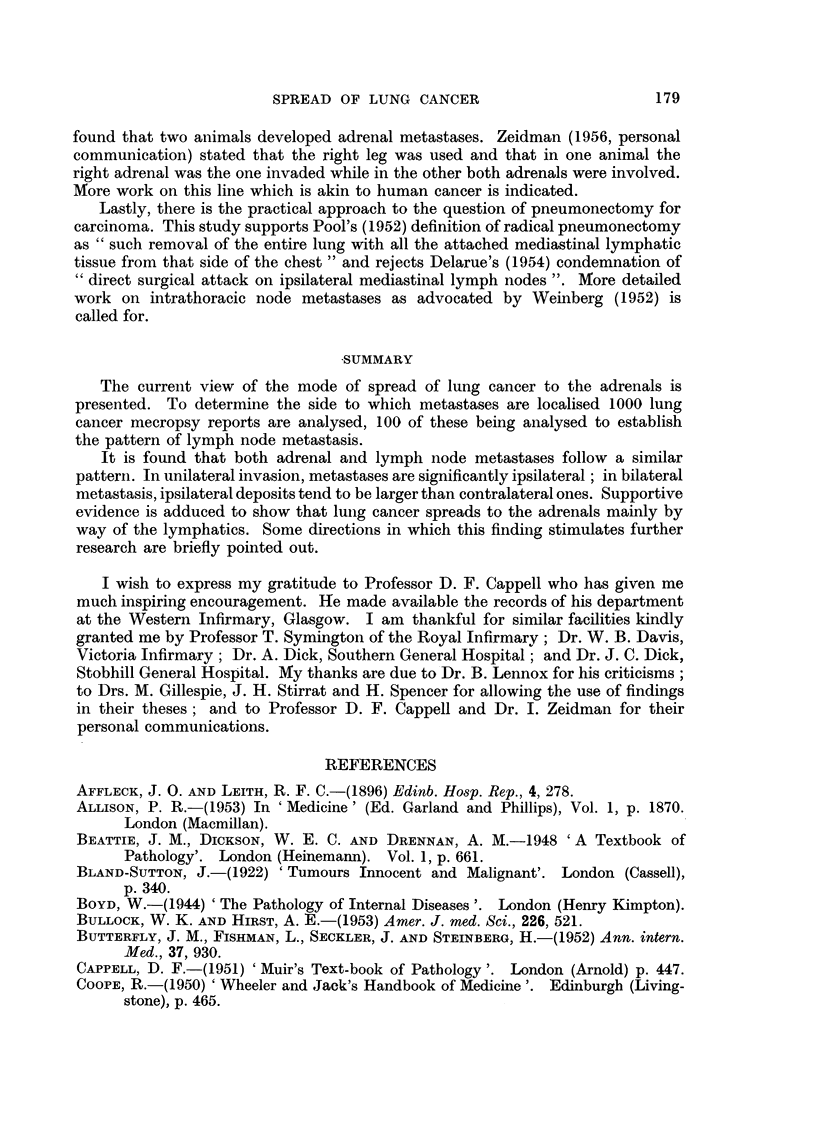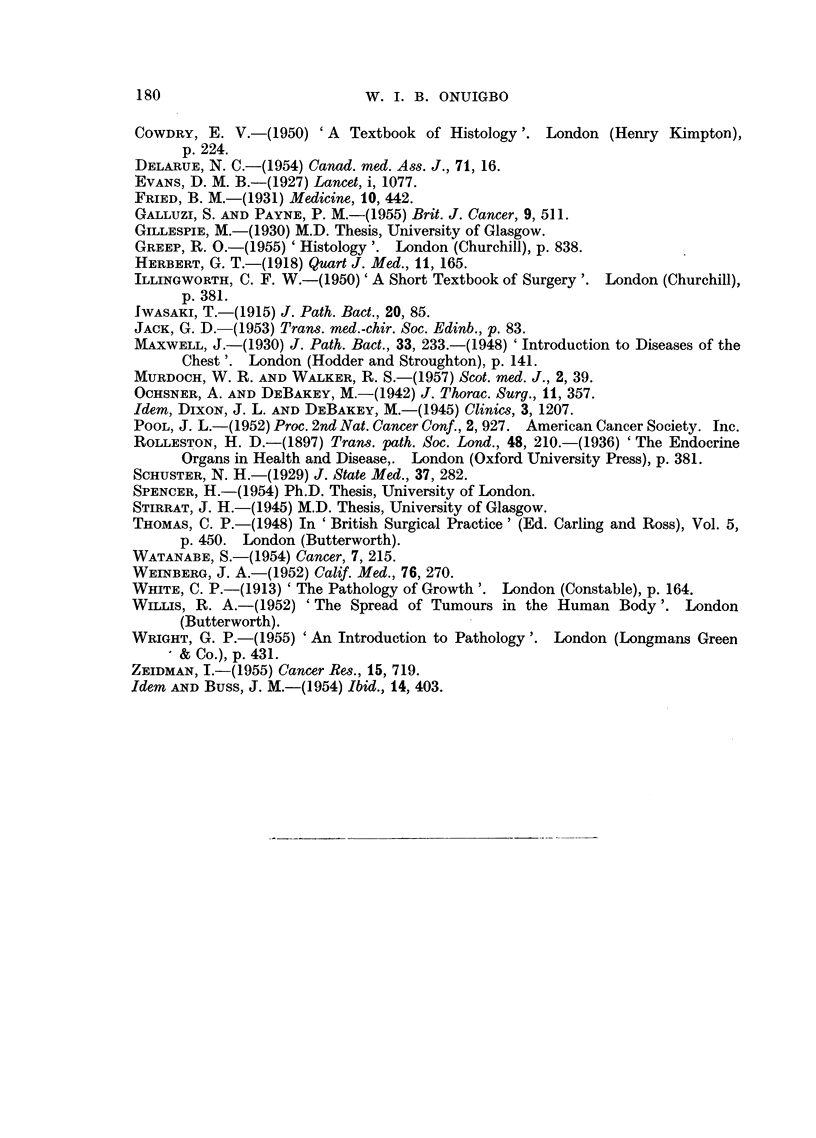# Some Observations on the Spread of Lung Cancer in the Body

**DOI:** 10.1038/bjc.1957.24

**Published:** 1957-06

**Authors:** W. I. B. Onuigbo


### SOME OBSERVATIONS ON THE SPREAD OF LUNG CANCER IN THE BODY

W. I. B. ONUIGBO

*From the Faculty of Medicine, University of Glasgow*

Received for publication April 4, 1957

THE advent of surgery in the treatment of bronchogenic carcinoma has encouraged, as Bland-Sutton (1922) forecast, a more detailed study of this fell disease. It has been observed that the adrenal is a common metastatic site (Cappell, 1951 ; Wright, 1955). A distant viscus like the adrenal is currently thought to be invaded via the blood stream (Willis, 1952). A few favour lymphatic spread to this organ in lung cancer—Herbert (1918), Maxwell (1930, 1948), Boyd (1944), and Coope (1950).

With regard to the accepted role of the systemic circulation in distant dispersal of malignant emboli, a puzzling finding has emerged : the higher incidence of adrenal metastases (around 33 per cent) as compared with renal secondaries (about 20 per cent) when account is taken of the relative weights and total blood supply of the two viscera. On account of this, Spencer (1954) concluded that " the adrenals either form a more suitable nidus for the survival of tumour emboli or, alternatively, that spread to the adrenals occurs through the lymphatic system ". Galluzi and Payne (1955), after noting this discrepancy, called for " a detailed study of the more obvious exceptions ".

TABLE I.—*The Disposition of* 163 *Unilateral Metastases of the Adrenals in* 1000 *Cases of Lung Cancer*

| Sources | Cases | Ipsilateral | Contralateral |
|---|---|---|---|
| Royal Infirmary (1940–56) | 277 | 26 | 21 |
| Western Infirmary (1940–56) | 266 | 28 | 11 |
| Stobhill General Hospital (1949–54) | 150 | 9 | 16 |
| Southern General Hospital (1950–56) | 130 | 10 | 8 |
| Victoria Infirmary (1945–55) | 77 | 13 | 2 |
| Stirrat (1945) | 75 | 8 | 4 |
| Gillespie (1930) | 25 | 6 | 1 |
| Totals | 1000 | 100 | 63 |

My interest in lung cancer was stimulated by the class in pathology as a result of which I embarked on a detailed study of necropsy records. This revealed that organs on the same side as the lung primary were usually the first, or only ones, to suffer metastasis. This paper presents observations which suggest that the high incidence of adrenal secondaries is explicable on the hypothesis of lymph-borne metastasis.

### *Method of study*

This study is based on 1000 lung cancer autopsies. Table I shows that 900 of these are histologically proven cases collected from the records of various

Glasgow Hospitals, and 100 are from the theses of Gillespie (1930) and Stirrat 1945). In every case showing unilateral adrenal deposits, it was noted whether the growth was ipsilateral or contralateral. For bilateral metastases, the side containing the larger deposit was similarly noted. Other visceral metastases were also recorded.

*Unilateral metastases*

In 1000 cases of pulmonary carcinoma, 100 ipsilateral adrenal metastases were encountered as opposed to 63 contralateral ones. The chi square test of significance shows that this preponderance of ipsilateral metastases is significant ($\chi_2 = 8{\cdot}4$, when $p < 0{\cdot}05$, for one degree of freedom).

*Bilateral metastases*

The issue of the place to be assigned to the arteries in the spread of cancer is fogged by the fact that in the majority of cases metastases are bilateral. However, it was observed from the descriptions that the growth on one side was often much larger than the other. In 63 such instances, the larger adrenal growth was found to be ipsilateral in 39 cases and contralateral in only 24. The numbers are small but it is thought that this phenomenon might be noted more often if its significance was realised. Gillespie (1930) remarked that the adrenals were " frequently both invaded, the one growth being larger than the other ". Beattie, Dickson and Drennan (1948) called attention to this manifestation in their Fig. 352 thus: " note the minute secondary growth in the opposite organ ". Cappell (1955, personal communication) has made this observation at autopsy. Case B 0158 of the Western Infirmary was an extreme example, for the ipsilateral adrenal weighted 500 g. and the contralateral only 40 g.

*Adrenal/renal relationship in metastasis*

The occurrence of bilateral adrenal metastases in combination with unilateral renal secondaries reveals a subtle way of assessing lymphogenous metastasis which will be brought out later. There were 32 such cases; as many as 22 showed invasion of the kidney on the ipsilateral side, the remaining 10 being contralateral. It may be stated also that of the total 71 unilateral kidney deposits found in the whole series, the kidney on the same side as the lung growth was invaded 46 times, the other kidney only 25. Western Infirmary Case A 6307 is of interest. A right (R.) lung primary spread to the liver and pancreas, to both adrenals and R. kidney, while in the brain there were deposits " in the R. temporal and parietal areas of the cortex; also in the dorsal part of the thalamus on the R. side, in the R. lateral lobe of the cerebellum and in the lower part of the pons, mainly on the R. side ".

*Location of the deposits*

Small metastases were most often described in the record books as having been found in the medulla. This is the common locus for early adrenal deposits (Rolleston, 1897; 1936). Malignant emboli might be expected to lodge in the cortex since the arteries end there as capillaries and do not reach the medulla. Willis (1952) explains the unexpected finding of medullary predominance on the basis of trans-capillary passage of tumour cells. It is necessary, however, to recognise that it is in the medulla that growths would develop first if retrograde lymphatic carriage of emboli occurred, because adrenal lymph vessels emerge from the medulla (Cowdry, 1950; Greep, 1955). Thus, the adrenals bear a striking resemblance to lymph nodes whose efferent vessels also emerge from the medulla. Consequently, by way of analogy, since secondary growths first develop in the medulla of a lymph node in retrograde metastasis (White, 1913; Zeidman, 1955), it is a simple deduction that in retrograde metastasis to the adrenal the growth should be first evident in the medulla. This conclusion is complementary to the findings of Bullock and Hirst (1953) who reported that their " histologic studies support the concept that intra-adrenal spread is primarily by lymphatic or tissue spaces but did not establish the mode of entry into the adrenals ". This picture was also obtained by Spencer (1954).

*Addison's disease*

There was only one case of Addison's disease in this series-(Royal Infirmary No. 167/1953). Long ago, Affleck and Keith (1896) concluded that this syndrome was " quite exceptional " in malignant disease; so did Butterfly *et al.* (1952) recently. This is not surprising when it is remembered that in about one-third of cases only one adrenal is invaded and even when both are involved it is only one of them—normally the ipsilateral one—that bears the brunt of malignant replacement, a replacement which further starts from within and encroaches progressively outwards.

*Centrifugal spread*

In the thousand cases under review, there were 426 (42·6 per cent) hepatic, 385 (38·5 per cent) adrenal and 173 (17·3 per cent) renal metastases. It is seen that this is in centrifugal order from the lung distalwards. The 4732 cases collected by Ochsner and DeBakey (1942) show the same trend. Indeed Fried (1931) has stated that " the frequency of metastases in bronchiogenic cancer in man diminishes with the distance from the central axis of the body ".

*Comparative study of lymph node metastases*

An analysis of 100 lung cancer necropsies recorded at the Western Infirmary from 1950 to 1952 was undertaken in order to determine the pattern of lymph node metastasis and to compare this with the pattern detailed above for adrenal metastases. First, lymph nodes were found to-be attacked in a centrifugal way, for intrathoracic nodes were involved in 81 cases, the upper abdominal nodes in 40 and lower abdominal in 7. This compares favourably with figures obtained by Ochsner, Dixon and DeBakey (1945). Both Evans (1927) and Willis (1952) confirm this mode of spread to lymph nodes in lung tumours. Secondly, the phenomenon known as " skipping " was observed 4 times. Here nodes are not involved in continuity but near-by nodes may be free while distant ones are attacked. Allison (1953) agrees with this finding. This is significant because it explains the possibility of similar skipping in the case of visceral deposits e.g. the invasion of the adrenal in the absence of hepatic secondaries. There is no need to go into the third feature which is the well-known retrograde metastasis. Fourthly, when node invasion was unilateral, the ipsilateral nodes suffered predominantly. Out of 42 such cases 41 were ipsilateral. Thomas (1948) and Jack (1953) support this finding. Fifthly, in bilateral lymph node metastasis, the growths on one side tend to be larger. This was so on the ipsilateral side in 8 out of the 11 cases so described. Schuster (1929) and Spencer (1954) made similar observations. Sixthly, when nodes on either side were involved but neither group was stated to be the larger, it was noted in 4 cases that more distant nodes were ipsilaterally invaded, thus simulating the relationship already noted in connection with bilateral adrenal and unilateral renal metastases occurring in the same subject at post mortem. The recent case reported by Murdoch and Walker (1957) illustrates this point, for the right lung primary, while metastasising to both groups of hilar nodes, replaced only right tracheal nodes. It is of interest to mention in passing that these findings are not peculiar to pulmonary carcinoma. For example, Illingworth's (1950) description of lymph node metastases in lingual carcinoma conveys the same topographical pattern.

### DISCUSSION

We have seen that both lymph-node and adrenal metastases show a common pattern of spread. While it is not intended to deny that cancer cells may spread by the systemic arterial route, the evidence now assembled clearly indicates that this mode of spread does not explain the observed facts in regard to adrenal metastases of bronchial carcinoma.

This conclusion should open up other lines for research. First, there is the corollary whether other viscera are invaded in a similar fashion. The method now advanced may prove useful in this respect not only with reference to bilateral organs such as the breast, but also in repect of unilateral viscera such as the caecum as well as midline structures such as the tongue which may bear lateral growths.

Secondly, what happens to tumour cells which are readily found in the pulmonary veins in human carcinoma of the lungs ? The experiments reported from Yale by Watanabe (1954) point to a possible clue. He found on introducing mouse bronchogenic carcinoma into the jugular veins of these animals that 160000 single viable tumour cells failed to yield metastases in contrast to those injected with clumped cells. Is this then the type of difference operative in systemic and lymphatic metastasis in man : the one turbulent and disruptive, the other quite the reverse ? Some other factor may be at work e.g. a possible difference in immunological potentiality between blood and lymph. Whatever may be responsible, if other investigators confirm that, despite easy access of tumour cells into systemic channels, lymphatic spread of carcinoma causes visceral metastases to a significant extent, then a search for the factors at work would seem to be called for. It may even be hoped that this may reveal a useful link in the chain of evidence being forged on the riddle of neoplasia.

The results hitherto obtained by means of animal experimental work might be vitiated by such unknown entities. Thus, is it an exact replica of the disease as it is met with in man to introduce malignant cells into the circulation of laboratory animals whose lymph nodes have not had the opportunity to function as the body's first line of defence ? In this respect, the approach of Iwasaki (1915) is noteworthy. He found that previous subcutaneous inoculation of tumour conferred on the animal a certain degree of immunity to subsequent intravenous injection of tumour cells. Furthermore, it is of interest that Zeidman and Buss (1954), after injecting tumour cells into a popliteal node afferent lymph vessel, found that two animals developed adrenal metastases. Zeidman (1956, personal communication) stated that the right leg was used and that in one animal the right adrenal was the one invaded while in the other both adrenals were involved. More work on this line which is akin to human cancer is indicated.

Lastly, there is the practical approach to the question of pneumonectomy for carcinoma. This study supports Pool's (1952) definition of radical pneumonectomy as " such removal of the entire lung with all the attached mediastinal lymphatic tissue from that side of the chest " and rejects Delarue's (1954) condemnation of " direct surgical attack on ipsilateral mediastinal lymph nodes ". More detailed work on intrathoracic node metastases as advocated by Weinberg (1952) is called for.

### SUMMARY

The current view of the mode of spread of lung cancer to the adrenals is presented. To determine the side to which metastases are localised 1000 lung cancer mecropsy reports are analysed, 100 of these being analysed to establish the pattern of lymph node metastasis.

It is found that both adrenal and lymph node metastases follow a similar pattern. In unilateral invasion, metastases are significantly ipsilateral ; in bilateral metastasis, ipsilateral deposits tend to be larger than contralateral ones. Supportive evidence is adduced to show that lung cancer spreads to the adrenals mainly by way of the lymphatics. Some directions in which this finding stimulates further research are briefly pointed out.

I wish to express my gratitude to Professor D. F. Cappell who has given me much inspiring encouragement. He made available the records of his department at the Western Infirmary, Glasgow. I am thankful for similar facilities kindly granted me by Professor T. Symington of the Royal Infirmary ; Dr. W. B. Davis, Victoria Infirmary ; Dr. A. Dick, Southern General Hospital ; and Dr. J. C. Dick, Stobhill General Hospital. My thanks are due to Dr. B. Lennox for his criticisms ; to Drs. M. Gillespie, J. H. Stirrat and H. Spencer for allowing the use of findings in their theses ; and to Professor D. F. Cappell and Dr. I. Zeidman for their personal communications.

### REFERENCES

AFFLECK, J. O. AND LEITH, R. F. C.—(1896) *Edinb. Hosp. Rep.*, **4**, 278.

ALLISON, P. R.—(1953) In ' Medicine ' (Ed. Garland and Phillips), Vol. 1, p. 1870. London (Macmillan).

BEATTIE, J. M., DICKSON, W. E. C. AND DRENNAN, A. M.—1948 ' A Textbook of Pathology'. London (Heinemann). Vol. 1, p. 661.

BLAND-SUTTON, J.—(1922) ' Tumours Innocent and Malignant'. London (Cassell), p. 340.

BOYD, W.—(1944) ' The Pathology of Internal Diseases '. London (Henry Kimpton).

BULLOCK, W. K. AND HIRST, A. E.—(1953) *Amer. J. med. Sci.*, **226**, 521.

BUTTERFLY, J. M., FISHMAN, L., SECKLER, J. AND STEINBERG, H.—(1952) *Ann. intern. Med.*, **37**, 930.

CAPPELL, D. F.—(1951) ' Muir's Text-book of Pathology '. London (Arnold) p. 447.

COOPE, R.—(1950) ' Wheeler and Jack's Handbook of Medicine '. Edinburgh (Livingstone), p. 465.

COWDRY, E. V.—(1950) ' A Textbook of Histology '. London (Henry Kimpton), p. 224.
DELARUE, N. C.—(1954) *Canad. med. Ass. J.*, **71**, 16.
EVANS, D. M. B.—(1927) *Lancet*, i, 1077.
FRIED, B. M.—(1931) *Medicine*, **10**, 442.
GALLUZI, S. AND PAYNE, P. M.—(1955) *Brit. J. Cancer*, **9**, 511.
GILLESPIE, M.—(1930) M.D. Thesis, University of Glasgow.
GREEP, R. O.—(1955) ' Histology '. London (Churchill), p. 838.
HERBERT, G. T.—(1918) *Quart J. Med.*, **11**, 165.
ILLINGWORTH, C. F. W.—(1950) ' A Short Textbook of Surgery '. London (Churchill), p. 381.
IWASAKI, T.—(1915) *J. Path. Bact.*, **20**, 85.
JACK, G. D.—(1953) *Trans. med.-chir. Soc. Edinb., p.* 83.
MAXWELL, J.—(1930) *J. Path. Bact.*, **33**, 233.—(1948) ' Introduction to Diseases of the Chest '. London (Hodder and Stroughton), p. 141.
MURDOCH, W. R. AND WALKER, R. S.—(1957) *Scot. med. J.*, **2**, 39.
OCHSNER, A. AND DEBAKEY, M.—(1942) *J. Thorac. Surg.*, **11**, 357.
*Idem*, DIXON, J. L. AND DEBAKEY, M.—(1945) *Clinics*, **3**, 1207.
POOL, J. L.—(1952) *Proc. 2nd Nat. Cancer Conf.*, **2**, 927. American Cancer Society. Inc.
ROLLESTON, H. D.—(1897) *Trans. path. Soc. Lond.*, **48**, 210.—(1936) ' The Endocrine Organs in Health and Disease,. London (Oxford University Press), p. 381.
SCHUSTER, N. H.—(1929) *J. State Med.*, **37**, 282.
SPENCER, H.—(1954) Ph.D. Thesis, University of London.
STIRRAT, J. H.—(1945) M.D. Thesis, University of Glasgow.
THOMAS, C. P.—(1948) In ' British Surgical Practice ' (Ed. Carling and Ross), Vol. 5, p. 450. London (Butterworth).
WATANABE, S.—(1954) *Cancer*, **7**, 215.
WEINBERG, J. A.—(1952) *Calif. Med.*, **76**, 270.
WHITE, C. P.—(1913) ' The Pathology of Growth '. London (Constable), p. 164.
WILLIS, R. A.—(1952) ' The Spread of Tumours in the Human Body '. London (Butterworth).
WRIGHT, G. P.—(1955) ' An Introduction to Pathology '. London (Longmans Green & Co.), p. 431.
ZEIDMAN, I.—(1955) *Cancer Res.*, **15**, 719.
*Idem* AND BUSS, J. M.—(1954) *Ibid.*, **14**, 403.